# Biventricular Takotsubo syndrome complicated with cardiogenic shock and ventricular septal rupture: a case report

**DOI:** 10.1093/ehjcr/ytae154

**Published:** 2024-03-22

**Authors:** Mariana Sousa Paiva, Sérgio Maltês, Catarina Brízido, Márcio Madeira, António Tralhão

**Affiliations:** Hospital de Santa Cruz, Centro Hospitalar de Lisboa Ocidental, Av. Prof. Dr. Reinaldo dos Santos, 2790-134 Carnaxide, Lisbon, Portugal; Hospital de Santa Cruz, Centro Hospitalar de Lisboa Ocidental, Av. Prof. Dr. Reinaldo dos Santos, 2790-134 Carnaxide, Lisbon, Portugal; Hospital de Santa Cruz, Centro Hospitalar de Lisboa Ocidental, Av. Prof. Dr. Reinaldo dos Santos, 2790-134 Carnaxide, Lisbon, Portugal; Hospital de Santa Cruz, Centro Hospitalar de Lisboa Ocidental, Av. Prof. Dr. Reinaldo dos Santos, 2790-134 Carnaxide, Lisbon, Portugal; Hospital de Santa Cruz, Centro Hospitalar de Lisboa Ocidental, Av. Prof. Dr. Reinaldo dos Santos, 2790-134 Carnaxide, Lisbon, Portugal

**Keywords:** Takotsubo syndrome, Ventricular septal rupture, Cardiogenic shock, Mechanical circulatory support, Case report

## Abstract

**Background:**

Takotsubo syndrome (TTS) mimics acute coronary syndromes but can lead to serious cardiac complications, emphasizing the need for improved understanding and management.

**Case summary:**

We describe a TTS case presented with cardiogenic shock due to ventricular septal rupture (VSR). Successful treatment involved mechanical circulatory support followed by VSR surgical closure.

**Discussion:**

Ventricular septal rupture is the rarest and deadliest complication associated with TTS. Prompt recognition and a multidisciplinary approach are crucial to achieve the best possible outcome.

**Learning points**

- Up to one in five patients diagnosed with Takotsubo syndrome may develop serious cardiac-related events.
- In such cases, immediate admission to a cardiac intensive care unit and the provision of adequate inotropic or mechanical circulatory support are essential for optimal patient care and outcomes.
- Ventricular septal rupture stands out as an exceptionally rare but highly lethal complication. Our findings highlight the role of surgical closure of the defect as the most effective available treatment option.

## Introduction

The typical clinical vignette of Takotsubo syndrome (TTS) is apical ballooning of the left ventricle (LV) in a middle-aged woman presenting with stress-elicited chest pain, after the exclusion of significant coronary artery disease.^1^ Well-known complications include acute heart failure and cardiogenic shock (CS), ventricular arrhythmias, LV outflow tract obstruction (LVOTO), and apical thrombus.^2^

We describe a case of TTS that developed CS associated with ventricular septal rupture (VSR), which was successfully treated with mechanical circulatory support (MCS) and VSR surgical closure.

## Summary figure

**Table:** 

Day 0	A 76-year-old woman presents to the emergency department (ED) with sudden onset of chest pain after the euthanasia of her cat.
	Electrocardiography (ECG) shows sinus tachycardia with ST-segment elevation in the anterolateral leads and pathologic Q-waves on V2–V4 leads. Emergent coronary angiography rules out obstructive coronary artery disease.
Day 1	Transthoracic echocardiogram (TTE) demonstrates severely reduced biventricular systolic function with apical ballooning, compatible with the diagnosis of TTS.
Day 5	Sudden SCAI-C CS managed with an intra-aortic balloon pump (IABP) and levosimendan. Absence of complications on repeated TTE.
Day 9	Shock resolution and IABP removal.
Day 11	Harsh pansystolic grade II/VI murmur at the lower left sternal border. Transthoracic echocardiogram shows an apical VSR causing a left-to-right shunt. Right-sided heart confirms a significant shunt with a Qp/Qs ratio of 2.3.
Day 26	Ventricular septal rupture surgical repair with a bovine pericardial patch.
Day 42	Hospital discharge after complete LV recovery.
5 months	Patient is asymptomatic and free of hospitalizations.

## Case presentation

A 76-year-old woman presented to the ED with sudden onset of chest pain and nausea after the euthanasia of her cat. Her medical history reported unmedicated hypertension and a previous depressive disorder. At presentation, her blood pressure was 149/105 mmHg, heart rate was 120 b.p.m., respiratory rate was 20 breaths/min, and oxygen saturation was 98% on room air. The remaining physical examination was unremarkable.

**Figure 1:**
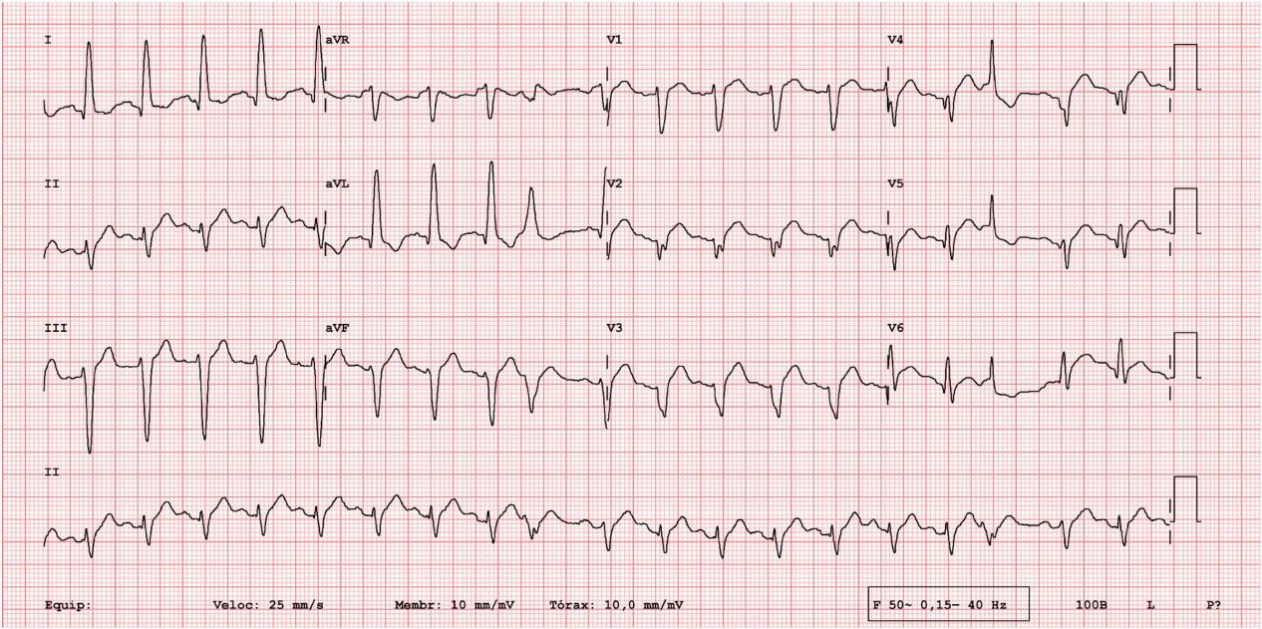
Twelve-lead electrocardiogram at admission showing sinus tachycardia with ST-segment elevation in the anterolateral leads and pathologic Q-waves on V2–V4 leads.

**Figure 2:**
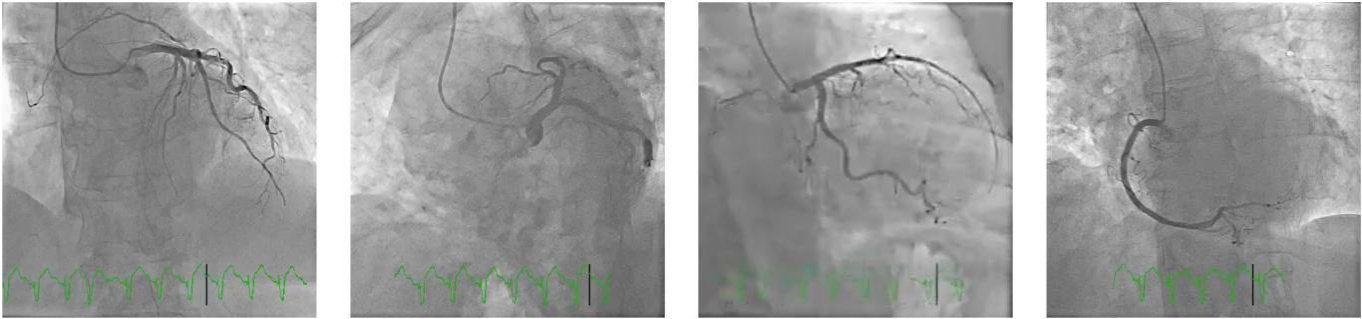
Coronary angiography at admission showing coronary arteries with minor irregularities but no significant stenosis.

Electrocardiography showed sinus tachycardia with ST-segment elevation in the anterolateral leads, pathologic Q-waves on V2–V4 leads, and a QTc interval of 401 ms (Fridericia formula, *Figure 1*). The patient was initially loaded with aspirin, clopidogrel, and heparin and underwent emergent coronary angiography, which ruled out obstructive coronary artery disease (*Figure 2*).

**Figure 3:**
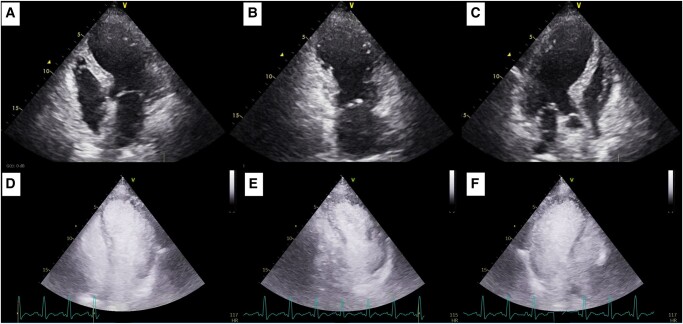
Transthoracic echocardiogram at admission exhibiting a non-dilated left ventricle with severely reduced ejection fraction (25%). (*A*) Apical four-chamber view. (*B*) Apical two-chamber view. (*C*) Apical three-chamber view. Transthoracic echocardiogram with SonoVue® contrast excluding left ventricular thrombus and rupture. (*D*) Apical four-chamber view. (*E*) Apical two-chamber view. (*F*) Apical three-chamber view.

Transthoracic echocardiogram demonstrated severely reduced LV ejection fraction (EF, 25%) with mid-to-apical hypokinaesia of all LV walls, apical ballooning, and no significant mitral regurgitation nor LVOTO (*Figure 3A–C*). The right ventricular (RV) longitudinal function was mildly reduced, and the apical free-wall segment was akinetic (see Supplementary material online, *Video S1*). Contrast TTE was performed to exclude LV thrombus and rupture (*Figure 3D–F*). Laboratory workup revealed elevated cardiac biomarkers with a characteristic dissociation between troponin and NT-proBNP elevations (peak high-sensitivity troponin T 2535 ng/L, peak NT-proBNP 18.900 pg/mL) and no other abnormalities.

Altogether, these findings supported the diagnosis of TTS, with an InterTAK score of 72 points (probability of TTS of 90.3%).^1^ The patient was admitted to the cardiac intensive care unit (CICU) and was started on neurohormonal blocking therapies for LV systolic dysfunction. Cardiac magnetic resonance imaging (CMR) was not performed owing to the patient’s claustrophobia.

Five days after admission, she progressed to SCAI-C stage, becoming more hypotensive, oliguric, and displaying hyperlactataemia. Repeated TTE did not show new findings. An IABP was implanted, and levosimendan was started, allowing haemodynamic recovery and IABP removal after 4 days.

**Figure 4:**
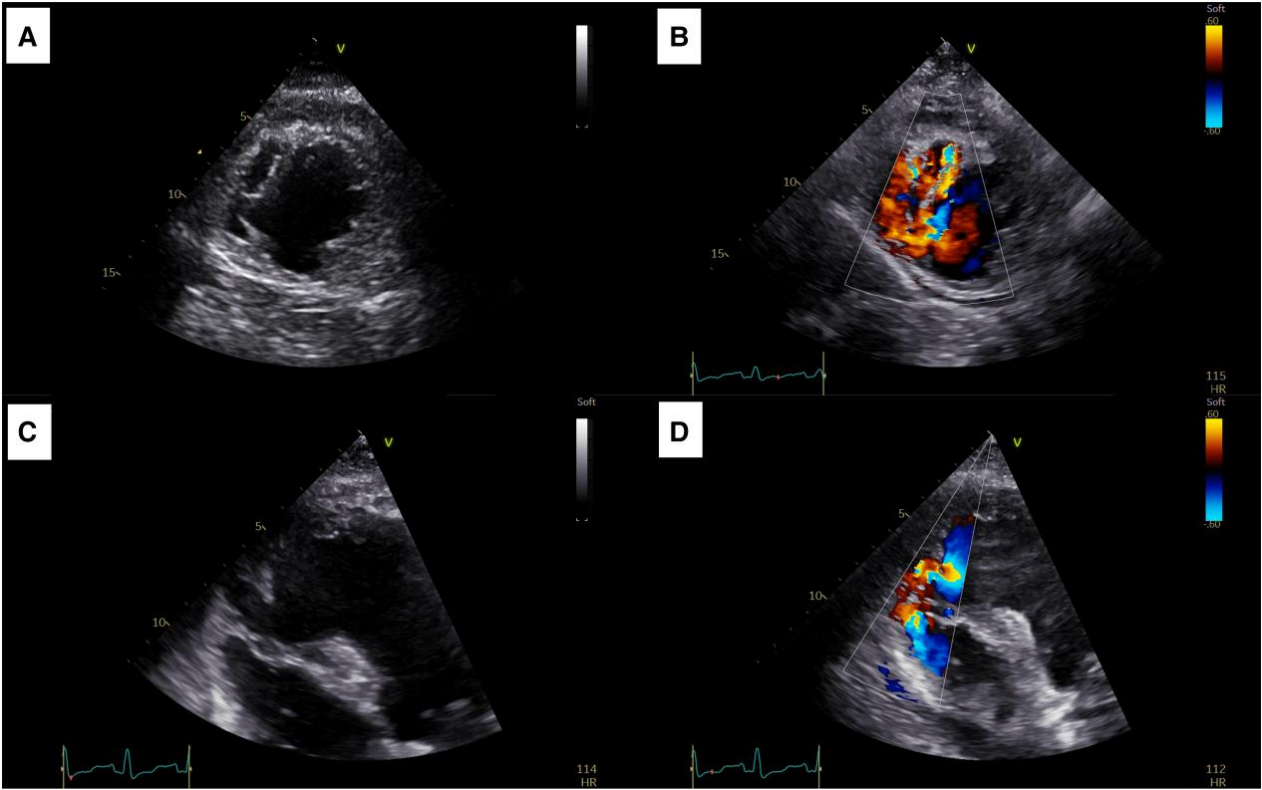
Transthoracic echocardiogram at Day 11 showing an apical ventricular septal rupture (∼19 mm) causing a left-to-right shunt (maximal gradient 60–65 mmHg). (*A*) Modified parasternal short-axis view at the apical level. (*B*) Modified parasternal short-axis view at the apical level with colour Doppler at the ventricular septal rupture location. (*C*) Modified apical four-chamber view. (*D*) Modified apical four-chamber view with colour Doppler at the ventricular septal rupture location.

On Day 11, a harsh pansystolic grade II/VI murmur was heard at the lower left sternal border. Although the LVEF had improved to 40%, an apical VSR (∼19 mm) causing a left-to-right shunt (maximal gradient 60–65 mmHg) was identified (*Figure 4*). Right-sided heart catheterization revealed a ‘step-up’ in oxygen saturation from the right atrium (53.4%) to pulmonary artery (82.3%). The pulmonary–systemic flow ratio (Qp/Qs ratio) was 2.3, consistent with a significant left-to-right shunt.

After heart team discussion, the patient was deemed a good candidate for surgical repair, based on her few comorbidities and intermediate surgical risk (EuroSCORE II 8%). Nonetheless, as she was haemodynamically stable, surgery was deferred for 14 days to allow better defect healing.

Twenty-six days after admission, the patient underwent surgery. *In vivo*, the 30 × 10 mm VSR was located at the mid-apical interventricular septum (IVS). The surgeon used a bovine pericardial patch to close the defect and was able to properly attach it as the surround myocardium was already fibrotic, without residual shunt (Qp/Qs pre-closure 2.3, Qp/Qs after closure 1).

**Figure 5:**
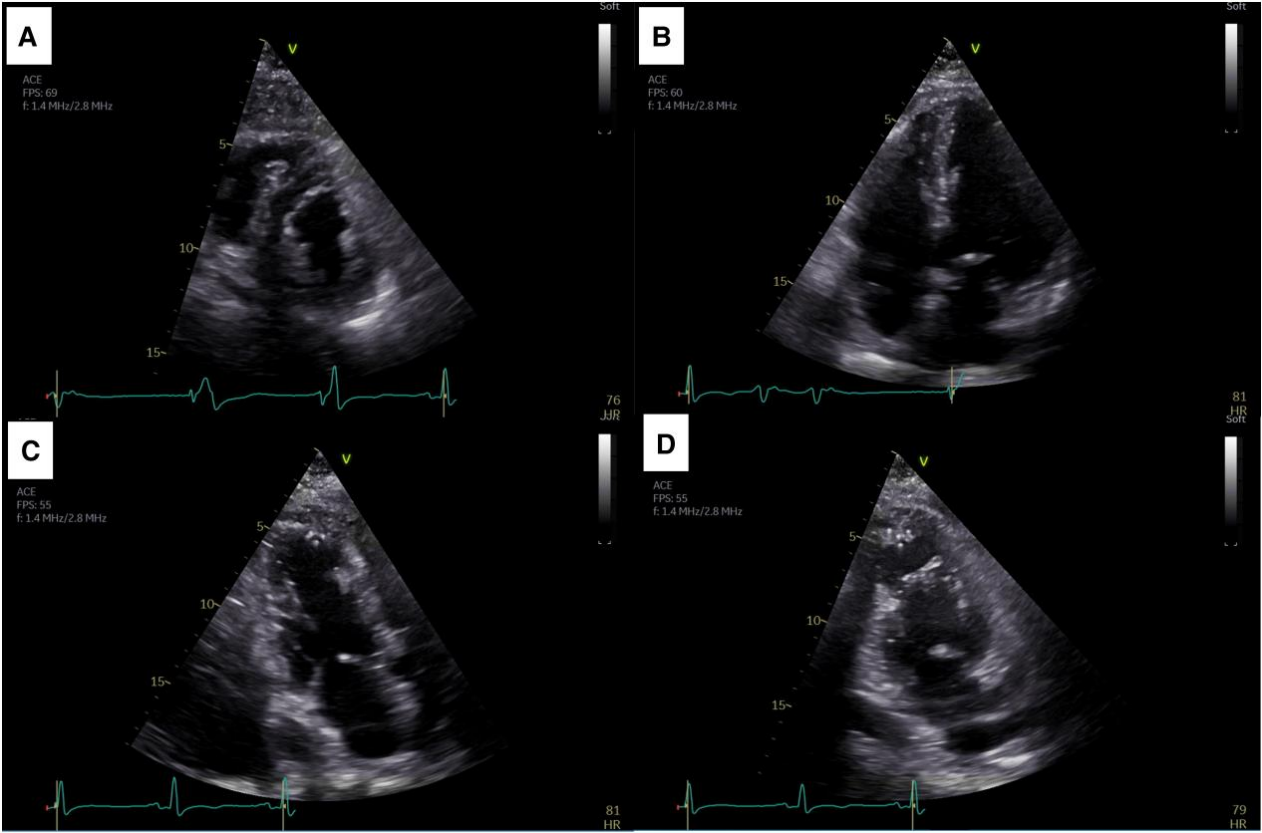
Transthoracic echocardiogram after surgery exhibiting the absence of residual ventricular septal rupture or left ventricular thrombus, and complete recovery of left ventricular ejection fraction to 55%, despite apical and mid-wall interventricular septum segment akinesia. (*A*) Parasternal short-axis view at the apical level. (*B*) Apical four-chamber view. (*C*) Apical three-chamber view. (*D*) Apical two-chamber view.

The post-operative period was uneventful, except for very frequent ventricular ectopic beats (>500/h) that were treated with bisoprolol and amiodarone. The TTE before discharge confirmed the absence of residual VSR and complete recovery of LVEF, despite apical and mid-wall IVS segment akinesia (*Figure 5*), and the ECG showed resolution of Q-waves on V2–V4 leads and only residual diffuse T-wave abnormalities (see Supplementary material online, *Figure S1*). The patient was discharged on post-operative Day 16.

At 5-month follow-up, the patient remained asymptomatic and free of hospitalizations.

## Discussion

Takotsubo syndrome represents ∼1–3% of all patients presenting to the ED with a possible ST-segment elevation myocardial infarction (MI).^2,3^ With increased recognition came the sense that this previously considered benign and transient entity could be associated with several life-threatening complications.^4^

In this case report, we describe the typical Takotsubo patient: a post-menopausal woman, with a previous psychiatric disorder, presenting with an ACS-like clinical picture after a significant emotional stress. After an initial coronary angiography showing non-obstructive disease, a myocardial infarction with nonobstrutive coronary arteries (MINOCA) diagnostic algorithm should be pursued. While CMR would have been a powerful tool to detect the extension of myocardial oedema, TTE alone, supported by the InterTAK criteria, allowed diagnostic confirmation.^5^

Despite initial clinical stability, there were some red flags at presentation, such as the persistent sinus tachycardia, highly elevated cardiac biomarkers, and the biventricular involvement with severe LV systolic impairment, that motivated close monitoring.^6^ Patients with a more severe clinical course that evolve into CS should be managed according to international guidelines but taking into consideration some specificities.^7^ Nowadays, it is well established that sympathetic stimulation plays a central role in TTS pathophysiology, as it induces microcirculatory dysfunction and direct cardiomyocyte toxicity.^6^ European consensus paper on TTS recommends avoiding catecholamines and preferring levosimendan. Mechanical circulatory support choice should be made according to patient severity and presence of LVOTO, but sooner than in other CS patients in order to avoid vasopressors.^8^

Left ventricular wall rupture is described in ∼1% of TTS patients and is associated with a very high mortality rate (75–80%), especially when affecting the LV free wall.^9,10^ The underlying mechanism is not yet completely understood. Histopathological analyses from prior studies consistently highlight features like inflammatory infiltration, transmyocardial myocardial necrosis with haemorrhage, and contraction band necrosis at rupture sites.^9^ Such pathological markers, including contraction bands, are not only characteristic of elevated catecholaminergic states but also reminiscent of post-MI changes.^11^ These observations suggest an extended or irreversible ischaemic pathway culminating in necrosis and rupture, potentially due to demand ischaemia fuelled by heightened adrenergic activity or intracardiac gradients.^12,13^ Concurrently, studies have linked elevated systolic and diastolic blood pressures and higher double products with VSR occurrences, underscoring the association between VSR and augmented LV intramural pressures and wall stress.^14^

According to Kumar *et al.*,^9^ the main risk factors for VSR are female gender, older age, higher arterial pressure, presence of ST-segment elevation in the inferior wall leads, and low LVEF. Zalewska-Adamiec *et al.*^10^ also suggest that a higher concentration of cardiac enzymes, higher GRACE scores, and a faster heart rate are important features.

Clinically significant uncorrected VSR originates from left-to-right blood shunting, causing RV pressure and volume overload, which, coupled with reduced systemic cardiac output ultimately, leads to the CS spiral, multiorgan failure, and death.

The definitive treatment of VSR almost always implies surgical closure, as a conservative approach, apart from anecdotal reports,^8^ is associated with a dire prognosis. Analogously to post-myocardial infarction (MI) VSR, the best timing to perform surgery is 14–21 days, in order to allow tissue healing.^15^ In TTS, this strategy is particularly useful as it allows the LV function to recover, reducing the overall risk of the procedure.^8^ During this period and if haemodynamic instability persists, treatment strategies include the use of inotropic support and MCS and the avoidance of vasopressors. Intra-aortic balloon pump provides modest circulatory support, reduces LV afterload, and therefore reduces left-to-right shunting. However, in more severe shock stages, other devices such as veno-arterial extracorporeal membrane oxygenation (VA-ECMO) and microaxial transaortic pumps may be necessary, at the expense of several serious complications. Finally, percutaneous defect occlusion may be a feasible alternative in smaller septal defects (<15 mm) and patients with prohibitive surgical risk, although its results are inferior when compared with surgery in the MI population.^16,17^

Due to the difficulty of performing clinical trials in these patients, observational studies and case reports such as this one may well be the best available evidence to help clinicians face a rare but frequently deadly complication.

## Lead author biography


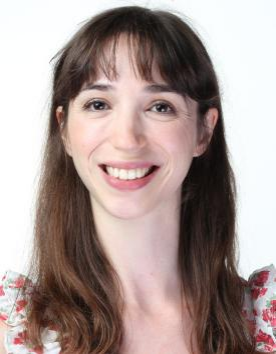


Mariana Paiva, MD, is a cardiology resident physician at Hospital de Santa Cruz, Centro Hospitalar Lisboa Ocidental EPE, Carnaxide, Portugal. She plans to pursue a career in clinical cardiology. Her research interests include heart failure, cardio-oncology, and adult congenital heart disease management.

## Supplementary Material

ytae154_Supplementary_Data

## Data Availability

The data underlying this article will be shared upon reasonable request to the corresponding author.

## Supplementary material

**Consent:** The authors confirm that written consent for submission and publication of this case report, including images and associated text, has been obtained from the patient in line with COPE guidance.

**Funding:** None declared.
