# Supplementary figures and images for: Biventricular Takotsubo syndrome complicated with cardiogenic shock and ventricular septal rupture: a case report
Source: Eur Heart J Case Rep. 2024 Mar 22;8(4):ytae154. doi: 10.1093/ehjcr/ytae154 (PMC10996925; doi:10.1093/ehjcr/ytae154)

# Supplemental Figure 1

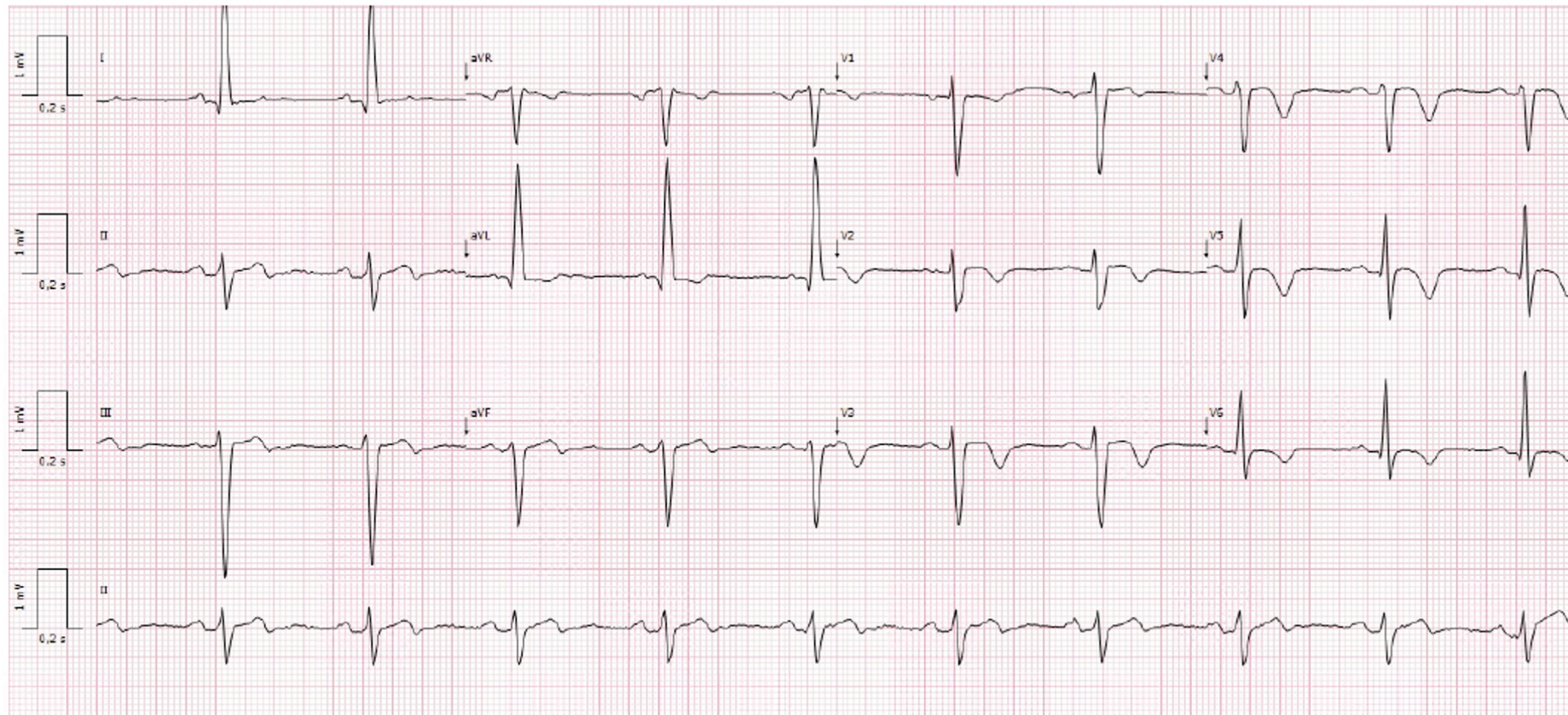

Supplement: ytae154_Supplementary_Data [file ytae154_supplementary_data.zip › Supplemental figure 1.pdf]
